# Assessment of Pain in Glaucoma Patients Undergoing Micropulse Transscleral Laser Therapy

**DOI:** 10.3390/jcm12072634

**Published:** 2023-03-31

**Authors:** Janrapee Sukkee, Natnaree Taechajongjintana, Kitiya Ratanawongphaibul, Rath Itthipanichpong, Anita Manassakorn, Visanee Tantisevi, Prin Rojanapongpun, Sunee Chansangpetch

**Affiliations:** 1 Department of Ophthalmology, Faculty of Medicine, Chulalongkorn University and King Chulalongkorn Memorial Hospital, Thai Red Cross Society, Bangkok 10330, Thailand; 2 Center of Excellence in Glaucoma, Chulalongkorn University, Bangkok 10330, Thailand

**Keywords:** glaucoma, laser, micropulse transscleral laser therapy, pain, cyclodestruction

## Abstract

Background: This study aimed to assess the pain experienced during micropulse transscleral laser therapy (MPTLT) and overnight thereafter and explore the factors associated with the pain. Methods: This prospective study included 100 eyes of 81 glaucoma patients undergoing MPTLT under retrobulbar anesthesia. All patients were asked to rate both types of pain using a numerical rating scale (NRS). The risk factors were explored using multivariable mixed-effects ordinal logistic regression. Results: The mean (SD) NRS pain score during the procedure was 3.57 (3.41) (range 0–10), which included no, mild, moderate, and severe pain in 30 (30%), 33 (33%), 17 (17%), and 20 (20%) eyes, respectively. The mean (SD) NRS score of overnight pain was 2.99 (2.28) (range 0–9), which included no, mild, moderate, and severe pain in 17 (17%), 59 (59%), 17 (17%), and 7 (7%) eyes, respectively. Twenty-seven (27%) eyes reported worse pain overnight than during the procedure. Increased age, initial intraocular pressure, and pain during the procedure were significantly associated with increased overnight pain (*p* < 0.05). Conclusions: Up to a fourth of eyes had worse pain after discharge. Older age, initial intraocular pressure, and pain during the procedure were risk factors for higher levels of overnight pain.

## 1. Introduction

Micropulse transscleral laser therapy (MPTLT) is one of the latest laser treatments for glaucoma. The mechanism of MPTLT involves the use of a diode laser to deliver repetitive short pulses of energy in an on-and-off manner. The ‘on’ mode involves the emission of an 810-nm-wavelength laser to produce thermal energy that is absorbed by the ciliary body, resulting in a decrease in aqueous production. The ‘off’ mode involves a resting period that allows the surrounding tissues to cool down to minimize complications from thermal energy. The use of MPTLT is increasing due to its good efficacy and safety profile [1,2].

MPTLT, as with conventional cyclophotocoagulation, is not a painless laser procedure and requires anesthesia. As MPTLT is a relatively new technology, there is no consensus on anesthesia guidelines. Thus, there have been several reports on local anesthesia practices, including the retrobulbar block [3], the peribulbar block [4], and analgosedation with topical anesthesia [5].

MPTLT, as an office-based procedure, has a number of advantages over operating room procedure in terms of convenience, cost, and efficiency [6,7,8]. Additionally, there is the advantage of avoiding the potential complications associated with general anesthesia and sedation. Regional anesthesia is required for office-based procedures performed on awake, nonsedated patients. Adequate pain control improves tolerance, procedural success, and patient satisfaction [9,10,11]. Conversely, inadequate pain management can lead to considerable anxiety. Patients who endure pain during or after the procedure may develop a fear of future treatment, which may dissuade them from undergoing subsequent interventions, potentially reducing treatment efficacy.

Despite receiving local anesthesia, some patients who undergo MPTLT report pain that might affect their quality of life and ability to perform activities of daily living. Few studies have evaluated pain due to MPTLT, and most have focused only on pain during the procedure [3,4,12,13,14]. This study aimed to assess the pain during the procedure as well as the overnight pain after MPTLT under retrobulbar anesthesia and explore the factors associated with the two types of pain.

## 2. Materials and Methods

This study was a non-comparative, prospective, and interventional trial. We enrolled all glaucoma patients scheduled for MPTLT at the King Chulalongkorn Memorial Hospital between March 2020 and March 2021. All participants were required to fulfill the following criteria: (1) 18 years old or older, (2) indicated or scheduled for MPTLT, (3) able and willing to attend follow-up appointments after the procedure, and (4) able to provide informed consent to participate in this study. Patients with painful blind eyes were excluded from the study.

This study was conducted in accordance with the tenets of the Declaration of Helsinki and approved by the local ethical committee, the Institutional Review Board of the Faculty of Medicine Chulalongkorn University, Bangkok, Thailand. Written informed consent was obtained from all study participants.

### 2.1. Procedure

Patients scheduled for MPTLT were premedicated with 50 mg of oral tramadol and 2 mg of oral diazepam. All patients received retrobulbar anesthesia with 2.5 mL of lidocaine hydrochloride 2% (Xylocaine^®^ 2%, Recipharm, Monts, France). The globes were assessed for akinesia 5 min after the injection. Rarely, a second injection of 1.5–2.5 mL lidocaine was administered if the globes did not exhibit akinesia. The total dose of lidocaine was less than 5 mL. Topical lidocaine hydrochloride 2% (Xylocaine^®^ Jelly 2%, AstraZeneca, Södertälje, Sweden) was administered subsequently, and experienced ophthalmologists performed MPTLT using the IRIDEX Cyclo G6^®^ Glaucoma Laser System with Micropulse P3^®^ Glaucoma device (IRIDEX Corporation, Mountain View, CA, USA) according to the manufacturer’s instructions. The laser settings were as follows: power, 2000 mW; duty cycle, 31.3%; and duration, 180 s for first-time MPTLT and 280 s for repeat MPTLT. The power was gradually decreased to 1800, 1500, and 1000 mW if the patient experienced unbearable pain. The laser probe was placed adjacent to the limbus and moved in a sliding motion along the sclera, sparing the 3 o’clock, 9 o’clock, and scleral thinning areas.

After the laser treatment, the operated eyes were treated with one drop of topical cyclopentolate hydrochloride 1% (Cyclogyl 1%, Alcon, Bornem, Belgium) and one drop of topical prednisolone acetate 1% (Pred Forte^®^, Allergan Pharmaceuticals Ireland, Westport, Ireland) and patched for 3 h. Paracetamol was administered as a “prn” prescription. All patients continued the same antiglaucoma medication that was taken before the procedure.

### 2.2. Data Collection

Patients undergoing MPTLT were asked to rate the “pain during the procedure” immediately after the procedure. The “overnight pain”, which was defined as early post-laser pain within 12 h after hospital discharge, was recalled and recorded during the first follow-up visit (day 1 post-operation). Both types of pain were recorded using the numerical rating scale (NRS), which ranged from 0 to 10. Jensen’s classification of pain was used to categorize the NRS score as no pain (NRS 0), mild pain (1–4), moderate pain (5–6), and severe pain (7–10) [15].

The data of individual participants, including age, sex, presence of diabetes mellitus, glaucoma type, inflammation, initial intraocular pressure (IOP), concurrent pilocarpine use, concurrent steroid use, repeated treatment, total energy, and total treatment area, were collected. The baseline IOP was measured using a Goldman applanation tonometer immediately before the laser treatment.

### 2.3. Statistical Analysis

Statistical analysis was performed using STATA 16.0 (Stata Corp., College Station, TX, USA). The mean and standard deviation of continuous variables are presented as descriptive statistics. Frequency and percentage are used to describe categorical variables. One-way analysis of variance (ANOVA) and chi-square tests were used to test the differences in clinical characteristics across pain levels. The association between the clinical characteristics and pain was further investigated using multivariable mixed-effects ordinal logistic regression for factors with *p*-values < 0.1 in univariable analysis. The NRS of “pain during the procedure” was included as a covariate in the “overnight pain” model. A *p*-value < 0.05 was considered statistically significant.

## 3. Results

A total of 100 eyes from 81 patients who underwent MPTLT were included in this study. No two eyes underwent MPTLT on the same day. The demographic and clinical characteristics of the patients are shown in Table 1. The mean score of pain during the procedure was 3.57 ± 3.41 (range 0–10). The mean score of overnight pain was 2.99 ± 2.28 (range 0–9). Overnight pain decreased significantly, with a mean difference of 0.75 compared with pain during the procedure (95% CI 0.11–1.39, *p* = 0.02). Eight patients did not complete the MPTLT procedure due to unbearable pain. 

Both pain during the procedure and overnight pain were classified into groups, as shown in Table 2. Comparing the two types of pain using Jensen’s classification, 31 (31%) eyes showed an improvement in the pain category, 42 (42%) eyes remained in the same category, and 27 (27%) eyes reported worsening pain.

The baseline factors, including age, sex, underlying diabetes mellitus, glaucoma type, inflammation, initial IOP, concurrent pilocarpine use, concurrent steroid use, repeated treatment, total energy, and total treatment area, were analyzed for their association with pain during the procedure; however, none showed statistical significance (all *p* > 0.10).

For the analysis of overnight pain, factors, including age, initial IOP, repeated treatment, total energy, treatment duration, and pain during the procedure, were included in the multivariable model. Given the collinearity between the total energy and treatment duration, independent models were used to analyze the two variables separately. Mixed-effects logistic regression showed that pain during the procedure, age, and initial IOP were significantly associated with the intensity of overnight pain, with higher values indicating greater severity (*p* < 0.05). The results of each model are shown in Table 3.

## 4. Discussion

In this study, 37 patients (37%) and 24 patients (24%) who underwent MPTLT reported moderate or greater pain during the procedure and overnight, respectively. We identified advanced age, high initial IOP, and high level of pain during the procedure as the factors associated with increased pain experienced overnight after the procedure.

MPTLT-related pain has been reported in the literature. Table 4 summarizes the study characteristics and the pain outcomes from five previous studies [3,4,12,13,14]. Tan et al. and Chang et al. demonstrated considerably lower prevalence of pain than Yelenskiy et al. and our study, whereas Preda et al. and Popa et al. reported mean visual analog scale values that were comparable to our mean NRS value. We hypothesize that the main difference in pain during the procedure between these studies was due to the total energy expended during the procedure, with higher energy resulting in greater pain. Among the studies using an average total energy greater than 130 mJ, our study found relatively lower levels of pain than others. Lower baseline IOP and a higher proportion of eyes with secondary glaucoma in our study may explain this finding. The association between initial IOP, type of glaucoma, and pain during MPTLT requires further study. It should be noted that the definition of pain and the pain measurement scale varied among studies.

Despite MPTLT being a non-incisional procedure, our study found that more than one-third of patients still experienced moderate to severe pain during the procedure. Popa et al. compared the pain experienced during MPTLT with that experienced during continuous wave cyclophotocoagulation (CW-CPC). Although they reported that the pain experienced during MPTLT was less than that during CW-CPC, the average pain score during MPTLT was as high as 6.02, which was considered as moderate pain [13].

According to our findings, 83% of patients experienced some degree of overnight pain, with 24% reporting moderate to severe pain. In addition, one-fourth of patients reported worse pain overnight than during the procedure. There were only two previous studies that reported the pain assessed at an early follow-up visit. The incidence ranged from 5.8% to 18.4%, and the degree of pain was mild. However, all previous studies utilized low total energy levels, whereas our study employed higher energy.

Overnight pain can develop after the effect of retrobulbar anesthesia has worn off. Possible contributors to overnight pain include intraocular inflammation, ciliary muscle spasm, and ocular surface pain. Lim et al. reported that 33.3% of patients who underwent MPTLT experienced postoperative anterior chamber inflammation [16]. Johnstone et al. demonstrated obvious ciliary muscle contraction during the application of MPTLT [17]. Keratitis, worsening of dry eye, and conjunctival laceration have been reported as complications of MPTLT [18]. The ocular surface is highly sensitive because of its dense sensory innervation [19]. Therefore, changes in ocular surface conditions can cause pain through altered pain receptor response and sensitivity. Notably, patients who reported no pain during the procedure can still experience severe overnight pain. Thus, MPTLT pain management should take into account the pain experienced during the laser treatment and that experienced overnight.

This study found that overnight pain was associated with advanced age, initial IOP, and level of pain during the procedure. Although aging is often related to an increase in pain threshold, several psychological investigations have revealed a decrease in pain tolerance and an increase in the duration of hyperalgesia after tissue injury in elderly individuals [20,21]. The fluctuation of IOP may be responsible for the positive relationship between the initial IOP and the degree of overnight pain. A sudden IOP rise is known to cause ocular pain in acute angle-closure crises. We speculate that individuals with high IOP may be more susceptible to the sudden IOP change following MPTLT. Thus, individuals with a higher baseline IOP may be more sensitive to pain than those with a lower IOP. Patients who experienced a high level of pain during the treatment were more likely to experience overnight pain. Therefore, pain assessment after the procedure is essential for guiding postoperative pain management. These factors could help identify individuals who are at risk of pain after discharge. Postoperative management should involve effective pain control strategies, such as a continuous anesthetic drug rather than a prn regimen, a strong cycloplegic agent, and adequate anti-inflammatory eye drops. Nonetheless, management should be tailored based on risks and benefits, with patients’ ocular and systemic medical conditions taken into account.

This was the first study to assess pain during the procedure and early postoperative pain after MPTLT as a primary objective. One limitation of this study was that all patients received retrobulbar anesthesia with a specific pre-medication pain control regimen before the laser treatment and one dose of cyclopentolate immediately after. Therefore, these results may not be generalizable to other anesthetic techniques or protocols. Second, pre-laser analgesic medication can affect the pain assessment. In this study, all patients received analgesics before the treatment, which should have theoretically reduced pain perception. Thus, the frequency and severity of pain in our study could have been underestimated. Third, the exact amount of lidocaine used for retrobulbar anesthesia was not recorded. The lidocaine dosage might influence the management of pain overnight. However, since the duration of action of lidocaine is less than two hours, it is less likely that its effect can alleviate pain throughout the night. We believe that our retrobulbar protocol, with the typical dose and short duration of action of lidocaine, had a lesser impact on the measurement of overnight pain. Fourth, overnight pain was recalled at follow-up and could have been affected by recall bias. Lastly, there was no exact dose or time when patients took the analgesics after the laser treatment.

In conclusion, pain due to MPTLT was frequent and could be severe both during the procedure and overnight thereafter. Thus, management for both types of pain should be seriously considered, especially in at-risk patients, such as elderly individuals, those with high IOP, and those who experienced a high level of pain during the procedure.

## Figures and Tables

**Table 1:** Baseline demographic and clinical characteristics and laser settings by pain categories.

		Pain during the Procedure					Overnight Pain				
	Total	No pain	Mild	Moderate	Severe	*p* Value	No Pain	Mild	Moderate	Severe	*p* Value
**No. of eyes**	100	30 (30%)	33 (33%)	17 (17%)	20 (20%)		17 (17%)	59 (59%)	17 (17%)	7 (7%)	
**Age (year)**	57.1 (16.3)	54.6 (17.5)	57.6 (16.4)	56.3 (19.0)	60.2 (12.9)	0.76	46.9 (14.0)	57.1 (16.6)	62.3 (16.4)	61.3 (13.0)	0.10
**Sex**						0.14					0.14 ^a^
Male	47 (58.0%)	15 (31.9%)	16 (34.0%)	4 (8.5%)	12 (25.5%)		9 (19.2%)	26 (55.3%)	9 (19.2%)	3 (6.4%)	
Female	34 (42.0%)	8 (23.5%)	12 (35.3%)	9 (26.5%)	5 (14.7%)		3 (8.8%)	25 (73.5%)	2 (5.9%)	4 (11.8%)	
**Diabetes mellitus**						0.49 ^a^					0.90 ^a^
No	70 (86.4%)	19 (27.1%)	26 (37.1%)	10 (14.3%)	15 (21.4%)		11 (15.7%)	44 (62.9%)	9 (12.9%)	6 (8.6%)	
Yes	11 (13.6%)	4 (36.4%)	2 (18.2%)	3 (27.3%)	2 (18.2%)		1 (9.1%)	7 (63.6%)	2 (18.2%)	1 (9.1%)	
**Intraocular pressure (mmHg)**	28.6 (11.9)	27.7 (10.9)	27.3 (12.2)	27.0 (11.0)	33.9 (13.6)	0.19	25.6 (10.1)	26.9 (10.8)	32.6 (14.1)	38.8 (13.4)	0.02
**No. of topical medications**	3.6 (0.8)	3.7 (0.9)	3.6 (0.7)	3.6 (0.6)	3.5 (0.8)	0.79	3.5 (0.9)	3.6 (0.6)	3.6 (0.6)	3.1 (1.5)	0.43
**Total energy (J)**	121.7 (27.1)	122.1 (29.6)	117.2 (24.0)	131.1 (29.4)	120.7 (25.8)	0.40	132.2 (35.7)	115.6 (20.3)	138.5 (31.8)	107.3 (17.0)	0.002
**Laser duration (second)**	196.4 (41.0)	198.0 (43.1)	190.5 (35.5)	209.4 (47.0)	192.8 (41.3)	0.47	216.5 (48.6)	186.2 (33.1)	221.2 (50.7)	177.9 (22.0)	0.002
**Diagnosis**						0.13					0.35
POAG	15 (15.0%)	6 (40.0%)	6 (40.0%)	1 (6.7%)	2 (13.3%)		4 (26.6%)	10 (66.7%)	0 (0.0%)	1 (6.7%)	
PACG	9 (9.0%)	3 (33.3%)	5 (55.6%)	1 (11.1%)	0 (0.0%)		1 (11.1%)	8 (88.9%)	0 (0.0%)	0 (0.0%)	
Secondary glaucoma	70 (70.0%)	21 (30.0%)	18 (25.7%)	13 (18.6%)	18 (25.7%)		11 (15.7%)	37 (52.9%)	16 (22.8%)	6 (8.6%)	
Childhood glaucoma	6 (6.0%)	0 (0.0%)	4 (66.7%)	2 (33.3%)	0 (0.0%)		1 (16.7%)	4 (66.6%)	1 (16.7%)	0 (0.0%)	
**First-time/repeat MPTLT**						0.95					0.02 ^a^
First-time	72 (72.0%)	21 (29.2%)	25 (34.7%)	12 (16.7%)	14 (19.4%)		9 (12.5%)	48 (66.7%)	9 (12.5%)	6 (8.3%)	
Repeat	28 (28.0%)	9 (32.1%)	8 (28.6%)	5 (17.9%)	6 (21.4%)		8 (28.6%)	11 (39.2%)	8 (28.6%)	1 (3.6%)	
**Eye inflammation**						0.92					0.23 ^a^
No cell in AC	58 (58.0%)	17 (29.3%)	21 (36.3%)	10 (17.2%)	10 (17.2%)		9 (15.5%)	36 (62.1%)	7 (12.1%)	6 (10.3%)	
Cell 1 + in AC	15 (15.0%)	6 (40.0%)	4 (26.7%)	2 (13.3%)	3 (20.0%)		5 (33.3%)	6 (40.0%)	4 (26.7%)	0 (0.0%)	
Cell 2 + in AC	27 (27.0%)	7 (25.9%)	8 (29.6%)	5 (18.6%)	7 (25.9%)		3 (11.1%)	17 (63.0%)	6 (22.2%)	1 (3.7%)	
**Laser power (mW)**						0.71 ^a^					0.30 ^a^
1000	1 (1.0%)	1 (100.0%)	0 (0.0%)	0 (0.0%)	0 (0.0%)		1 (100.0%)	0 (0.0%)	0 (0.0%)	0 (0.0%)	
1500	2 (2.0%)	0 (0.0%)	2 (100.0%)	0 (0.0%)	0 (0.0%)		0 (0.0%)	1 (50.0%)	0 (0.0%)	1 (50.0%)	
1800	1 (1.0%)	0 (0.0%)	1 (100.0%)	0 (0.0%)	0 (0.0%)		0 (0.0%)	1 (100.0%)	0 (0.0%)	0 (0.0%)	
2000	96 (96.0%)	29 (30.2%)	30 (31.3%)	17 (17.7%)	20 (20.8%)		16 (16.7%)	57 (59.4%)	17 (17.7%)	6 (6.2%)	
**Area of treatment (degree)**						0.55 ^a^					0.30
180	3 (3.0%)	0 (0.0%)	2 (66.7%)	0 (0.0%)	1 (33.3%)		0 (0.0%)	3 (100.0%)	0 (0.0%)	0 (0.0%)	
270	17 (17.0%)	4 (23.5%)	7 (41.2%)	1 (5.9%)	5 (29.4%)		3 (17.6%)	8 (47.1%)	4 (23.5%)	2 (11.8%)	
300	2 (2.0%)	1 (50.0%)	1 (50.0%)	0 (0.0%)	0 (0.0%)		2 (100.0%)	0 (0.0%)	0 (0.0%)	0 (0.0%)	
360	78 (78.0%)	25 (32.0%)	23 (29.5%)	16 (20.5%)	14 (18.0%)		12 (15.4%)	48 (61.5%)	13 (16.7%)	5 (6.4%)	
**Concurrent pilocarpine use**						0.67 ^a^					0.41 ^a^
No	99 (99.0%)	29 (29.3%)	33 (33.3%)	17 (17.2%)	20 (20.2%)		16 (16.2%)	59 (59.6%)	17 (17.2%)	7 (7.0%)	
Yes	1 (1.0%)	1 (100.0%)	0 (0.0%)	0 (0.0%)	0 (0.0%)		1 (100.0%)	0 (0.0%)	0 (0.0%)	0 (0.0%)	
**Concurrent steroid use**						0.63					0.65 ^a^
No	72 (72.0%)	20 (27.8%)	25 (34.7%)	11 (15.3%)	16 (22.2%)		12 (16.7%)	42 (58.3%)	12 (16.7%)	6 (8.3%)	
Yes	28 (28.0%)	10 (35.7%)	8 (28.6%)	6 (21.4%)	4 (14.3%)		5 (17.9%)	17 (60.7%)	5 (17.9%)	1 (3.5%)	

Data shown as mean (standard deviation) and n (%); MPTLT, micropulse transscleral laser therapy; POAG, primary open angle glaucoma; PACG, primary angle closure glaucoma; AC, anterior chamber; ^a^ Fisher’s exact test.

**Table 2:** Categories of pain during the procedure and overnight.

		Overnight Pain				
		No Pain	Mild Pain	Moderate Pain	Severe Pain	Total
**Pain during** **procedure**	No pain	12	16	1	1	30
	Mild pain	3	21	8	1	33
	Moderate pain	1	12	4	0	17
	Severe pain	1	10	4	5	20
	Total	17	59	17	7	100

**Table 3:** Multivariable mixed-effects ordinal logistic regression showing the factors associated with overnight pain.

	Model 1			Model 2		
	Odds Ratio	95% CI	*p*-Value	Odds Ratio	95% CI	*p*-Value
**Pain during procedure**	1.30	1.14 to 1.49	<0.001 *	1.30	1.13 to 1.49	<0.001 *
**Age**	1.03	1.01 to 1.06	0.019 *	1.03	1.01 to 1.06	0.019 *
**First-time/Repeat MPTLT**						
First-time	reference			reference		
Repeat	1.00	0.23 to 4.30	0.998	1.02	0.22 to 4.73	0.976
**Intraocular pressure**	1.04	1.01 to 1.08	0.023 *	1.04	1.01 to 1.08	0.023 *
**Total energy**	1.00	0.99 to 1.01	0.717	-	-	-
**Duration of laser treatment**	-	-	-	0.99	0.98 to 1.01	0.707

MPTLT micropulse transscleral laser therapy; * denotes *p* < 0.05.

**Table 4:** Literature on micropulse transscleral laser therapy-related pain.

Study	Diagnosis	Mean Age (year)	Mean Initial IOP (mmHg)	Total Energy (J)	Anesthesia	Pain during the Procedure	Pain after the Procedure
Tan AM et al., 2010 [4]	SCG (NVG + others): 50% POAG: 22.5% PACG: 25% Juvenile glaucoma: 2.5%	63.2 ± 16.0	39.3 ± 12.6	62.50	Peribulbar	- 26.3%: tolerable pain without a need for additional anesthesia - 5.3%: pain required additional regional anesthesia	Pain on the first day - 18.4%: mild, tolerable pain not requiring use of oral analgesia - 0%: moderate, tolerable pain with regular usage of oral analgesia - 0% severe, pain despite regular dosing of oral analgesia
Chang HL et al., 2021 [12]	POAG: 100%	65.0 ± 15.8	27.8 ± 7.6	100.00	Retrobulbar	- 7.7%: mild, tolerable pain without a need for topical analgesia - 3.8%: moderate, tolerable pain with the use of topical analgesia - 0%: severe, intolerable pain even with the use of topical analgesia	Early postoperative pain at follow up examination - 5.8% mild pain
Yelenskiy A et al., 2018 [14]	POAG: 88% SCG (NVG): 5% Others: 7%	73.0 ± 12.0	21.5 ± 9.0	112.68–150.24	Retrobulbar/peribulbar	- 63% reported pain	Pain during the immediate postoperative period - 45% reported pain
Preda MA et al., 2020 [3]	OAG: n/a% PXG: n/a% Inactive NVG: n/a%	62.6	39.1 ± 13.8	100.16–162.76	Retrobulbar	- Mean VAS = 5.86	Pain beyond the day of the procedure - none
Popa G et al., 2019 [13]	n/a	66	44.2 ± 10.7	100.16–150.24	Retrobulbar	- Mean VAS = 6.02	n/a
Current study	POAG: 15% PACD: 9% SCG (NVG + others): 70% Childhood glaucoma: 6%	57.1 ± 16.3	28.6 ± 11.9	112.68–175.28	Retrobulbar	- Mean NRS = 3.57 - 33%: mild pain, NRS 1-4 - 17%: moderate pain, NRS 5-6 - 20%: severe pain, NRS 7-10	Early post laser pain within 12 h - Mean NRS: 2.99 - 59%: mild pain, NRS 1-4 - 17%: moderate pain, NRS 5-6 - 7%: severe pain, NRS 7-10

IOP, intraocular pressure; POAG, primary open-angle glaucoma; OAG, open-angle glaucoma; SCG, secondary glaucoma; NVG, neovascular glaucoma; PXG, pseudoexfoliation glaucoma; PACG, primary angle-closure glaucoma; PACD, primary angle-closure disease; VAS, visual analog scale; NRS numerical rating scale; n/a, data not available.

## Data Availability

Data available on request due to restrictions (privacy and ethical).
